# Beyond Behavior: Linguistic Evidence of Cultural Variation in Parental Ethnotheories of Children’s Prosocial Helping

**DOI:** 10.3389/fpsyg.2020.00307

**Published:** 2020-03-12

**Authors:** Andrew D. Coppens, Anna I. Corwin, Lucía Alcalá

**Affiliations:** ^1^Education Department, University of New Hampshire, Durham, NH, United States; ^2^Anthropology Department, Saint Mary’s College of California, Moraga, CA, United States; ^3^Department of Psychology, California State University, Fullerton, CA, United States

**Keywords:** ethnotheory, prosocial helping, socialization, parenting, culture, toddlers, linguistic

## Abstract

This study examined linguistic patterns in mothers’ reports about their toddlers’ involvement in everyday household work, as a way to understand the parental ethnotheories that may guide children’s prosocial helping and development. Mothers from two cultural groups – US Mexican-heritage families with backgrounds in indigenous American communities and middle-class European-American families – were interviewed regarding how their 2- to 3-year-old toddler gets involved in help with everyday household work. The study’s analytic focus was the linguistic form of mothers’ responses to interview questions asking about the child’s efforts to help with a variety of everyday household work tasks. Results showed that mothers responded with linguistic patterns that were indicative of ethnotheoretical assumptions regarding children’s agency and children’s prosocial intentions, with notable contrasts between the two cultural groups. Nearly all US Mexican-heritage mothers reported children’s contributions and participation using linguistic forms that centered children’s agency and prosocial initiative, which corresponds with extensive evidence suggesting the centrality of both children’s autonomy and supportive prosocial expectations in how children’s helpfulness is socialized in this and similar cultural communities. By contrast, middle-class European-American mothers frequently responded to questions about their child’s efforts to help with linguistic forms that “pivoted” to either the mother as the focal agent in the child’s prosocial engagement or to reframing the child’s involvement to emphasize non-help activities. Correspondence between cultural differences in the linguistic findings and existing literature on socialization of children’s prosocial helping is discussed. Also discussed is the analytic approach of the study, uncommon in developmental psychology research, and the significance of the linguistic findings for understanding parental ethnotheories in each community.

## Introduction

A surge in research on children’s prosocial motivation and development over the last two decades has deepened understanding of very young children’s abilities and apparent inclinations to help and assist others. It is increasingly clear that toddlers from a number of communities are able to notice when help is needed in standardized research tasks and offer assistance voluntarily (Warneken, 2015). The strength and apparent universality of this pattern in toddlers has supported the suggestion that early prosociality may be “rooted” in innate biological tendencies (Warneken and Tomasello, 2009; Callaghan and Corbit, 2018), toward, for example, social affiliation, emotional coregulation, and sharing goals.

However, more research is needed on the ecological realities of children’s everyday helping to understand whether and how children extend their early prosocial abilities and inclinations into culturally relevant and socially complex forms of prosocial behaviors (note House et al., 2013). Evidence that toddlers are *able* to be helpful in standardized research tasks is important but does not address whether toddlers are *likely* to do so at home or whether their bids for involvement are met with opportunities that support the development of practices in which children learn to contribute and collaborate. Prosocial helping in these contexts closely relates to direct and indirect caregiver structuring efforts (Kärtner et al., 2010; Köster et al., 2016; Giner Torréns and Kärtner, 2017), as well as to the cultural values and assumptions that give them meaning.

A number of researchers have called for an increased focus on everyday contexts of children’s development (Hedegaard, 2009; Dahl, 2017; Rogoff et al., 2018). Overall, evidence is needed to understand how individual and community processes are linked in everyday practices to provide insights beyond mere variable-based descriptions of behavior (Rogoff, 2003). Psychological research on children’s development has increasingly recognized the limitations of approaches that overlook cultural and socialization processes, as well as the importance of cross-cultural evidence to understand how variation in prosocial behavior might be tied to socialization practices (Köster et al., 2015; Nielsen et al., 2017; Brady et al., 2018).

When the everyday contexts of children’s helping are in focus, important questions emerge regarding children’s prosocial development. For example, recent laboratory-based research has found that children younger than age 2 can help *voluntarily* and that requests for their assistance or incentives may undermine how and whether they help (Warneken and Tomasello, 2008, 2013, 2014; Hepach et al., 2012). Yet, middle-class European-heritage families commonly use incentives, requests, and task assignments at younger ages (Dahl, 2015; see also Waugh et al., 2015) and at older ages when middle-class children in various communities help both minimally and reluctantly (Ochs and Kremer-Sadlik, 2013; Coppens et al., 2016). What views, assumptions, and cultural values guide the deployment of these socialization practices when they may be unnecessary or even counterproductive to children’s prosocial development? In communities where such practices are less common and children’s voluntary contributions to everyday household work are more common (see Paradise and Rogoff, 2009), what views and values relate to different socialization processes and different prosocial outcomes?

Common experimental tasks in recent research on young children’s prosocial helping present young children with situations in which the child’s help is *needed* for an adult to successfully complete their work, a central aspect of some definitions of prosociality (Dunfield et al., 2011). However, a frequent if not predominant pattern of everyday helping at home entails young children getting involved with work that parents are already accomplishing without difficulty. Often, children’s involvement may be instrumentally *un*helpful at the outset (see Hammond and Brownell, 2018). To understand how prosocial helping interfaces with children’s patterns of involvement in everyday activities at home, we must ask: How do parents understand and respond to young children’s curiosity or interest in taking part or helping, even when those bids risk slowing things down and may require parents to accomplish the work at hand while also guiding children? A long-standing speculation in research on young children’s prosocial helping suggests that when this pervasive “unhelpfulness” is met with parenting practices that preclude children from taking part in everyday work, children’s motivation to help prosocially may gradually diminish (Rheingold, 1982). Support for children regularly observing and taking part in everyday work appears to vary considerably across cultural communities, with middle-class children commonly prevented from doing so (Morelli et al., 2003; Rogoff, 2014).

Cultural perspectives in research on children’s helping emphasize the embeddedness of children’s prosocial development in the cultural values and socialization practices of their families and communities (e.g., Giner Torréns and Kärtner, 2017), a need to consider forces in play at both the “roots” (i.e., developmental origins) and “branches” (i.e., developmental trajectories) of children’s prosocial development (Hay, 2009). Evidence of striking cultural differences in older children’s prosocial helpfulness (e.g., Ochs and Izquierdo, 2009; Alcalá et al., 2014; Coppens et al., 2016) suggests that cultural values and socialization practices are important for young children’s prosocial development and vary considerably across communities.

This study demonstrates that linguistic analyses can provide insights into how and why parents from different cultural communities interact with their children to shape the contexts and trajectories of their prosocial development. Inquiry into individuals’ cultural perspectives can be methodologically challenging, as views, values, and cultural assumptions are often held and enacted implicitly. In this study, we show that parents’ perspectives on children’s helping can be studied by examining *ideational* features of interview data or “how individuals represent themselves and others as (inter)acting in the world” (Konopasky and Sheridan, 2016, p. 5). We document linguistic patterns that characterize *how* parents in different cultural communities report their children’s help, as evidence by variation in assumptions regarding children’s motivations and abilities, their learning and developmental processes, and parents’ own roles in guiding children to learn to help and collaborate.

Parental ethnotheories are culturally patterned views, values, and assumptions that function as local guiding frameworks for understanding patterns of child behavior and guiding parents’ approaches to caregiving and socialization. In this study we focus on parental ethnotheories related to children’s efforts and motivation to help in everyday household work, a prominent venue for prosocial learning and development. Parental ethnotheories constitute one of three subsystems of the “developmental niche” or cultural context of children’s development – the others include “the physical and social settings of everyday life” and “the customary practices of child care” (Harkness et al., 2010, p. 77). The developmental niche framework theorizes the cultural organization of child development settings by, in part, accounting for cultural values and ideas that inform parents’ structuring of settings they consider optimal for raising their children (e.g., Harkness and Super, 1992; Parmar et al., 2004).

This study’s linguistically oriented analysis of parental ethnotheories regarding children’s prosocial helping provides insight into the question: Why do children in societies all over the world readily and willingly contribute to everyday family work by middle childhood, yet children in many middle-class, postindustrial communities do not? Parental views and assumptions about children’s helpfulness may be central to explaining this pattern. Indeed, despite the worldwide prevalence of voluntary, prosocial helping among young children, “many middle-class parents in the United States view [children helping out without prompting] as impossible,” an unrealistic expectation (Ochs and Kremer-Sadlik, 2015b, p. 95).

Below, we review evidence on parental ethnotheories common to the two cultural communities included in the present study: middle-class European-heritage communities and indigenous or indigenous-heritage communities of Mexico and the Americas.

Research describes two prominent themes in the ethnotheories of parents and families of the middle class or what is often referred to in the literature as WEIRD backgrounds – i.e., Western, educated, industrialized, rich, and democratic (Henrich et al., 2010). On the one hand, numerous studies highlight children’s personal autonomy and independence as cultural values and parental socialization goals that are definitional to “child-centered” middle-class cultural models of parenting. German, Dutch, and European-American parents of middle-class backgrounds showed high preferences for independence-oriented socialization goals and practices (Harkness et al., 2000; Keller et al., 2006, 2010). Kusserow’s (2004) ethnographic study of individualisms in parental ethnotheories in several New York City communities found that “by age three Parkside [a community of largely white, affluent, highly educated families] children were already considered little competitors – small but complete ‘little people’ with their own tastes, desires, needs, and wants” (p. 81). Especially as children enter school, self-expression and children’s competitive pursuit of preferences and desires are supported as developmental ends in and of themselves (Bellah et al., 2008), with parents socializing children for “movement through achievement space” (Gee et al., 2001).

On the other hand, considerable research describes many middle-class parents’ orientations toward children’s development as a parent-controlled endeavor. Middle-class parents in postindustrial communities are highly and intently involved in the organization and management of nearly all aspects of children’s everyday lives, a paradigm that Lareau (2003) has called “concerted cultivation” and that LeVine et al. (1994) characterize as a “pedagogical” model of child development and good parenting (see also Popkewitz, 2003). This ethnotheoretical orientation may include implicit assumptions that the “engine” of children’s growth and development is parental effort to not only organize but also motivate and incentivize children through enriching and self-enhancing developmental curricula at home, school, and numerous extracurricular activities. This adult-managed characteristic of middle-class childhoods suggests a hierarchical relation between adults’ requests of and directives toward children and children’s compliance in taking part – two separate systems.

Contradiction between independence values and parental control in middle-class cultural models of parenting and schooling has long been acknowledged. Whiting (1978) described a “dependency hang-up,” which Weisner (2001) found to be prominent across over 18 years of longitudinal observation with United States middle-class families of varying lifestyles. The contradiction also pervades a number of middle-class institutions designed for children’s learning and development. For example, Tobin (1995) observes that the early childhood education settings of many middle-class communities at once value children’s “authentic” or “free” expression of emotions but permit such expression only within imposed, highly scripted, and normative models of speech, interaction, and emotional experience. In a detailed ethnographic study of middle-class family life in contemporary Los Angeles (see Ochs and Kremer-Sadlik, 2013), these contradictions were pervasive:

the much-championed ideology that children, at least by school age, should be relatively self-reliant was rarely apparent in children’s behavior in CELF households… Many of these middle-class parents struggled with the potentially unwanted consequences of investing in their child as the center of their attention and energy. They worried that promoting children’s self-absorption inhibits their self-sufficiency and attunement to helping others in their surroundings. (Ochs and Kremer-Sadlik, 2015a, p. 744)

Deeper understanding of how middle-class parents balance or negotiate these practice-embodied ethnotheoretical currents in everyday interaction and activity in the home may be key to understanding a *developmental niche* that allows for waning prosociality in middle-class European-heritage children’s everyday household participation.

Research with indigenous and indigenous-heritage communities in Mexico and the United States show that parental ethnotheories are closely linked to children’s inclusion in family and community life as well as to cultural expectations of children’s prosocial contributions. Gaskins (1999a) highlights three “principles of engagement” organizing Mayan children’s learning and development and the cultural ethnotheories that relate to them. Although certainly not a characteristic of all indigenous communities, the principles have resonance with a wide range of similar cultural groups (Paradise and Rogoff, 2009; Lancy et al., 2010).

First, many parents of indigenous communities of the Americas structure children’s everyday lives to emphasize the *primacy of “adult” activities* in children’s learning (Gaskins, 1999a). Children’s full integration in productive activities is valued specifically for children learning to help and collaborate (Rogoff, 2014). Age is seldom a requirement for helping; as soon as infants are able to sit on their own they can start to observe others work, and as soon as they can walk they can start to help (Alcalá and Cervera Montejano, in preparation). Mazahua (indigenous, in Mexico) toddlers are often present when their mothers sell produce at the local market, and they are given opportunities to learn the ways mothers negotiate with clients and organize their produce stand even as they play (Paradise, 1994). In a Maya community in Chiapas, when 2-year-olds attempt to enter into work activities, adults “orient and reorient the activity” to facilitate children’s participation, respecting and acknowledging their “agency in these coparticipatory interactions” (Martínez Pérez, 2015, p. 113). For example, a 21-month-old boy took initiative to help his grandmother shell beans, approaching her as she started the activity. The grandmother took this opportunity to show him how to sort the good pods from the rotten ones, guiding his attention to important details of the activity using repetition as the child imitated actions, and “…contributing to competence development without dissuading the child from taking on an agentive role” (p. 127).

Second, particular *ideas about children’s learning and development* inform how many indigenous-heritage American parents support children’s helping in everyday settings (Gaskins, 1996, 1999a). Yucatec Maya parents view development, including prosocial helping, as intrinsic to children and as unfolding gradually and naturally (Gaskins, 1999b, p. 110). Nonetheless, expectations for children’s responsibility “circulate” in a variety of ways. In a Tz’utujil Maya community of Guatemala, parents communicate clear expectations that children attend and observe the work and other social activity going on around them, verbally correcting inattention, with the aim of children learning to pitch in to help (Chavajay, 1993). For many Mexican and Mexican-heritage mothers, having access to work and other mature activities allows children to become *acomedido/a* – attentive and responsive to when help is needed (López et al., 2015). Being *acomedido/a* is regarded as a vital cultural value and socialization goal for children – some parents state that helping only when asked has no merit (Alcalá et al., 2018). Maya mothers’ ethnotheories of development are also grounded in rituals and cultural practices that indirectly guide children’s development, such as the *hetzmek’* ceremony (i.e., Maya baptism) at around 4 months of age (Cervera Montejano, 2007). In this ceremony, the infant is presented with tools to help them be productive and with foods such as pumpkin seeds to help them be more intelligent, increase their memory, and be eloquent and “*alegre*.” Development of intelligence or “understanding” – *na’at* in Maya – is described as “remembering their responsibility.” Although expectations of responsibility are common among indigenous-heritage communities of the Americas, contingent *assignment* of chores or other responsibilities is rare (Coppens et al., 2016). “Too much teaching” is viewed as inefficient and motivationally distracting for socializing children’s contributions (de Haan, 1999; see also Gaskins, 1999a; Cervera, 2016).

Third, in many indigenous-heritage American communities, the *independence of children’s prosocial motivation* is both a goal of socialization and a cultural assumption of children’s early social dispositions (Gaskins, 1999a). According to Yucatec Maya parental ethnotheories, when children have opportunities to observe others work, they become motivated (*se animan*) to learn to help (Alcalá and Cervera Montejano, in preparation; see also Paradise, 2005). Instead of attempting to motivate children’s prosocial helping directly, young children attempting to help are often strategically rejected, as a way to provoke them and increase their autonomous motivation, awareness of others, and sense of responsibility. As toddlers insist on pitching in, despite rebuffs, adults reorient the activity breaking it down into a sequence that allows the child’s involvement (Martínez Pérez, 2015). In a Quechua community in Peru, Bolin (2006) reports that “children are treated with respect and allowed to develop at their own pace, largely in accordance with their inclinations” (p. 152; see also Alcalá and Jiménez Balam, 2015). Respect is the catalyst that allows children to develop a sense of responsibility toward their family and community. Forcing or obligating a child to help is considered inappropriate in many indigenous American communities, as it may restrict the child’s “development of understanding” or create family animosity, disrupting the social fabric that supports children’s development (Alcalá and Cervera Montejano, in preparation; Bolin, 2006). Chavajay and Angelillo (2014) note that there is no word for “control” in Tz’utujil Maya that relates to parenting practices; instead, the word “guidance” is common along with “respect,” which are long-term developmental goals that apply to both parents and children.

In any community, language plays a key role in connecting idealized values and developmental goals with the everyday practices that shape children’s prosocial helping and development. The present study draws on linguistic anthropological perspectives in the traditions of Heath (1983), Schieffelin and Ochs (1986a, b), Goodwin (1990), Wortham (2001a), and Goodwin and Cekaiate (2018) – among many others – to develop insights regarding the cultural and parental ethnotheories that may guide parents’ socialization of children’s prosocial helping, and may help to explain cultural variation in the trajectories of children’s prosocial development from toddlerhood to middle childhood.

We illustrate the potential of linguistic analyses for understanding cultural variation in parental ethnotheories of children’s prosocial development with data from interviews with mothers of toddlers from two cultural backgrounds: US Mexican-heritage families with background in indigenous-heritage cultural practices and middle-class European-American families with extensive schooling background over several generations. The interviews asked mothers about their child’s help in everyday household work and the ways that children usually became involved, and our analyses examine how mothers report their children’s involvement. This linguistic analysis leverages methodological strengths of two common approaches to studying parental ethnotheories: asking parents explicit questions about their views (see Coppens and Rogoff, in preparation) and examining naturally occurring talk among family members interacting at home (e.g., Ochs and Kremer-Sadlik, 2015a). Reflexive conversational interviews in this study created a shared topical focus designed to solicit and evoke parents’ views on children’s helping, and our analyses center on implicit features of language-in-use, where “danger that the linguistic means are consciously manipulated with respect to social desirability is practically negligible” (Keller et al., 2004, p. 294).

## Materials and Methods

### Participants and Communities

Participants were mothers from two cultural communities who had a 2- to 3-year-old child – 20 US Mexican-heritage mothers and 20 middle-class European-American mothers, living near Monterey and San Francisco bay areas of California. The US Mexican-heritage mothers’ toddlers averaged 2.6 years (9 girls, 11 boys), and the middle-class European-American mothers’ toddlers averaged 3.2 years (10 girls, 10 boys). All families averaged two household members under 18 years old and two household members 18 years old or older (usually, two parents).

We refer to the two communities as US Mexican-heritage Background in Indigenous Ways (BIW) and European-American Extensive Schooling Experience (ESE). These labels are intended to denote entire constellations of cultural values and practices, rather than single variables such as “indigeneity” or “education attainment,” which fits with a way of theorizing cultural phenomena as situated within dynamic, historically significant cultural *paradigms* (Rogoff et al., 2014; Dayton and Rogoff, 2016). Our emphasis in sampling decisions was families’ likely participation in such paradigms, although practical concerns preclude testing this experience comprehensively.

In the US Mexican-heritage BIW families, parents averaged 9.2 grades of schooling completed and grandparents averaged 4.6 grades. This level of schooling is consistent with communities in Mexico and the United States that have some historical continuity with indigenous communities of Mexico (Bonfil Batalla, 1996; Rogoff et al., 2014). In addition, families’ regional backgrounds were in Mexican states with strong indigenous histories, including rural areas of Michoacán, Oaxaca, and Jalisco. Most parents worked in agricultural harvesting and packing, service-sector jobs, or construction. In the European-American ESE families, parents averaged 17.0 grades of schooling completed and grandparents averaged 16.0, characteristic of a cultural group usually referred to as middle class (Bronfenbrenner et al., 1996; Lareau, 2000). All parents in this community were born in the United States. Most parents worked in education, in business-sector jobs, or in healthcare.

### Interviews

Mothers participated in one 45- to 60-min semistructured interview conducted conversationally, in the language and location of the mother’s choosing, without their children. Most (88%) of the US Mexican-heritage BIW mothers chose to be interviewed in Spanish; all of the European-American ESE mothers’ interviews were in English. In each community, about half of the interviews were in the family’s home and half at a public park. A bilingual Mexican-heritage female research assistant who was blind to the study’s hypotheses led the interviews, and the first author, male and also bilingual, routinely and systematically added a few conversational or clarification questions. In each interview, one of the researchers shared the mother’s ethnicity.

Mothers were introduced to the study during recruitment and on the consent form simply as an investigation of how children help around the house. The session began with casual inquiry about the child’s school and afterschool activities and the family’s weekend plans, as well as questions regarding family composition, home languages, and parents’ occupations. (Further demographic information was requested at the end of the interview.)

The conversational interview included open-ended (e.g., In a normal day, does your child help around the house? How? What do they do?) and semistructured questions regarding the kinds of things the 2- to 3-year-old helped with around the house. All mothers were asked whether and how voluntarily the child helped with a scripted list of household tasks, which was the focus of our analysis in this study. In this scripted list, there were 27 tasks, some that benefit the whole family (e.g., washing family dishes or clothes, sweeping the kitchen, taking out the trash) and some related to the child’s personal things and spaces (e.g., putting away their toys, making their bed). Questions were omitted if mothers had reported the task in prior portions of the interview.

Protocols for participant recruitment, interviews, and data security and confidentiality were approved by the University of California, Santa Cruz Institutional Review Board. All research participants gave written informed consent in accordance with the Declaration of Helsinki.

### Coding

We coded exchanges between the interviewer and the mother regarding questions about children’s helping that used a specific “child-as-agent-helping” form, for example: “Does your child help with washing the dishes?” This linguistic form positions the child as the subject and agent and positions helping as the object of the child’s actions. Because the scripted list of household tasks was asked conversationally, the child-as-agent-helping questions were asked in both complete and abbreviated versions. For example, following the mother’s response to the above question about the dishes, the interviewer might have asked, “And, what about helping with putting clean dishes away?” or “How about setting the table?” Abbreviated questions were only included in the analysis when they closely followed a complete child-as-agent-helping question.

Our analyses focused on the linguistic form of mothers’ responses to child-as-agent-helping questions. We identified three main ways that mothers in each community responded:

(1) The mother’s response maintains linguistic *continuity with the child-as-agent-helping* form. Even simple “yes” or “no” responses to questions such as, “Does your child help with taking out the trash?,” would be coded here. Parent responses may indicate “we” if the activity is commonly done together or is collaborative, for example: “Sure, Jonah sometimes helps with the dishes when we have some time in the morning to wash them.”

The next two codes describe two kinds of “pivots” in the linguistic features of mothers’ responses.

(2) The mother’s response *pivots to a caregiver-as-agent* linguistic form. A mother may pivot to position *herself* as the agent in response to a question about whether *the child* helped with washing clothes, for example noting that she does not “have” the child get involved with the work or that she has not “let” the child do that type of work.
(3) The mother’s response *pivots to non-helping as the object of the child’s actions*. For example, the mother may report the child “liking” to play with water or being “interested” in brooms. This is not the same as liking to help or being interested in some activities over others. The response suggests a view that the child is, for example, playing and not helping or does not either care about or understand the idea of helping with work that is taking place.

Coding was conducted primarily by the first author, bilingual, and a native English speaker. Half (50%) of the interviews in Spanish (in the US Mexican-heritage BIW community) were independently coded by the third author, bilingual, and a native Spanish speaker. Differences in coding were few, and all were resolved via discussion.

## Results

**FIGURE 1:**
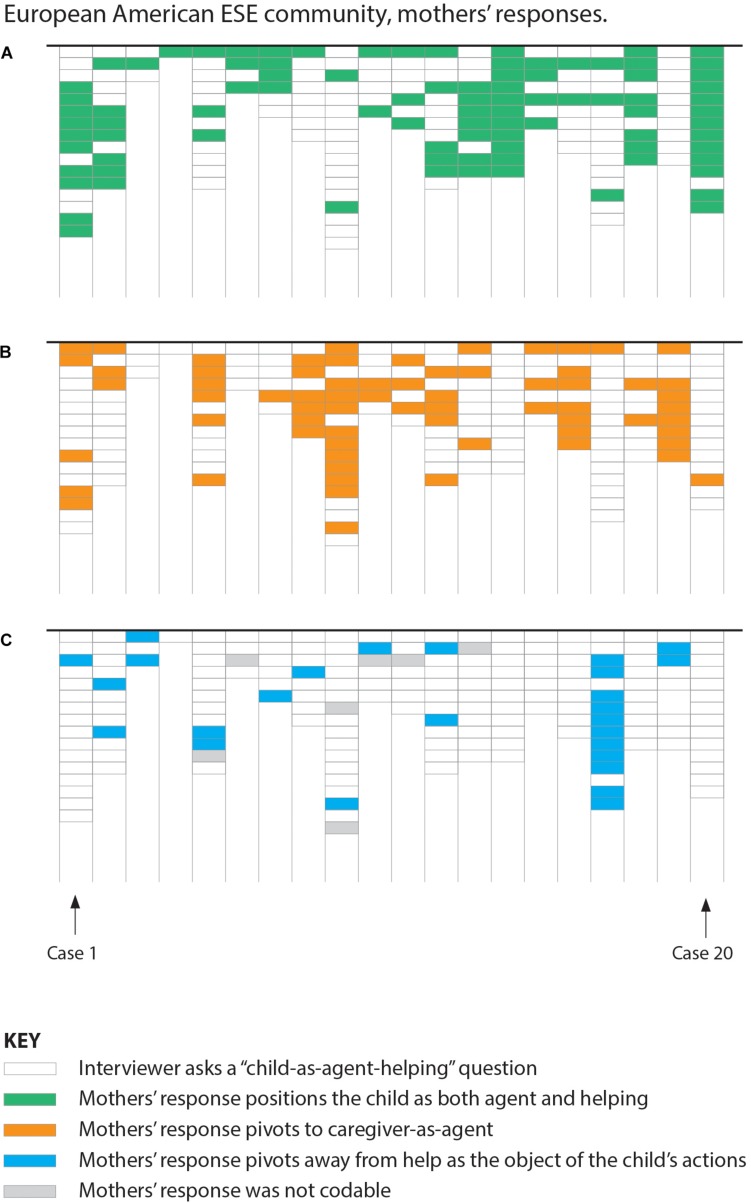
Case graph showing European-American ESE mothers’ responses. Panels **(A–C)** should be read as “layers” which separately show each type of coded response (color-filled cells) against the overall number of interview prompts (no-fill cells). Vertical columns represent coding for one mother, disaggregated by coding type across panels **(A–C)**. Cells represent one coded response to a “child-as-agent-helping” interview question.

**FIGURE 2:**
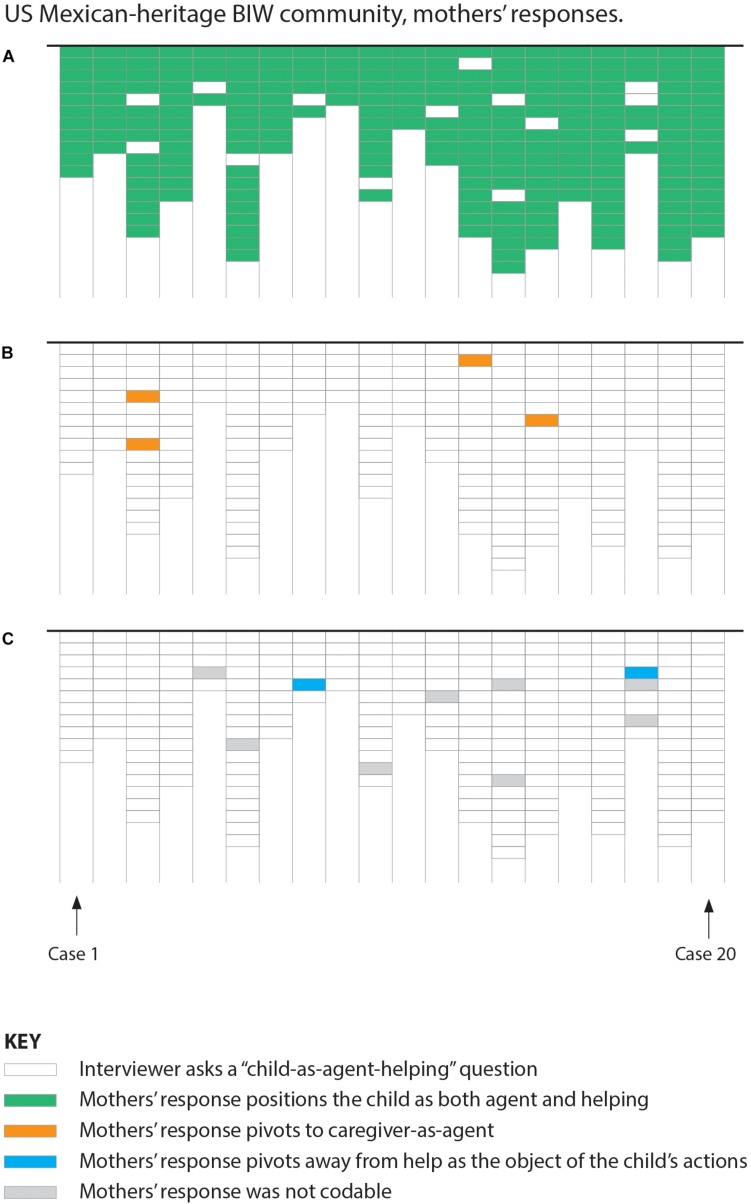
Case graph showing US Mexican-heritage BIW mothers’ responses. Panels **(A–C)** should be read as “layers” which separately show each type of coded response (color-filled cells) against the overall number of interview prompts (no-fill cells). Vertical columns represent coding for one mother, disaggregated by coding type across panels **(A–C)**. Cells represent one coded response to a “child-as-agent-helping” interview question.

Overall, across all 20 mothers’ interviews in each community, 191 child-as-agent-helping questions were asked among the European-American ESE families (average of 9.6 questions per mother), and 247 such questions were asked among the US Mexican-heritage BIW families (average of 12.4 questions per mother). Figures 1, 2 show within- and between-community patterns in mothers’ responses at two units of analysis: patterns at the level of linguistic utterance (i.e., the figure “cells”) and at the level of individual mothers (i.e., the figure “columns”). Table 1 summarizes the findings quantitatively, at both levels of analysis.

**TABLE 1:** Summary of within-group patterns and between-group cultural contrasts in responses to child-as-agent interview questions among US Mexican-heritage BIW and European-American ESE mothers.

		Mothers responding with a linguistic “pivot”		
	# of mothers	Any Pivot	Caregiver-as-agent Pivot	Away-from-help Pivot
US Mexican-heritage BIW	20	5 (25%)	3 (15%)	2 (10%)
		**	**	*
European American ESE	20	17 (85%)	16 (80%)	12 (60%)

		**Responses with a linguistic “’pivot”**		
	**# of interview prompts**	**Any Pivot**	**Caregiver-as-agent Pivot**	**Away-from-help Pivot**

US Mexican-heritage BIW	247	6 (2%)	4 (2%)	2 (1%)
		**	**	**
European American ESE	191	94 (49%)	68 (36%)	26 (14%)

The upper two rows show summaries with individual mothers as the unit of analysis. The lower two rows show summaries with single utterances (i.e., an interview question prompt with the mothers’ response) as the unit of analysis. Statistical significance of tests for cultural differences is indicated with ** p < 0.001 and * p < 0.01 and was conducted with Barnard’s exact test.

With linguistic utterances as the unit of analysis, cultural-group comparisons showed significant differences in response patterns. In the European-American ESE community, 49% (94/191; see Figures 1B,C) of the child-as-agent-helping questions were followed by a “pivot” of some kind, whereas in the US Mexican-heritage BIW community, this was the case for only 2% (6/247; see Figures 2B,C) of the child-as-agent-helping questions, *p* < 0.001, Barnard’s exact test (BET; Barnard, 1945; Mehta and Senchaudhuri, 2003). Cultural-group differences in proportions of utterances were also statically significant regarding each of the two types of pivots, as indicated in Table 1.

With individual mothers as the unit of analysis, cultural-group comparisons also showed significant differences. In the European-American ESE community, 85% (17/20; see Figures 1B,C) of mothers responded with at least one pivot during their interviews, compared to 25% (5/20; see Figures 2B,C) of mothers in the US Mexican-heritage BIW community, *p* < 0.001, BET. Cultural-group differences in proportions of mothers were also statically significant regarding each of the two types of pivots, as indicated in Table 1.

### Linguistic “Pivots,” Common Among Mothers in a Middle-Class European-American Community

In this section, we provide several contextualized examples of the linguistic “pivot” patterns that were common in the European-American ESE mothers’ speech. Our purpose in examining such patterns was to develop insights into mothers’ assumptions about children’s helping at the level of cultural ethnotheories, providing clues about the cultural paradigms that inform prosocial socialization strategies that structure the child’s immediate environment.

Below, we illustrate a common pattern in the European-American ESE mothers’ speech with the following exchange between interviewers (I-1 is the primary interviewer; I-2 is the secondary interviewer and first author) and one mother, which begins with a follow-up question to an earlier question about helping:


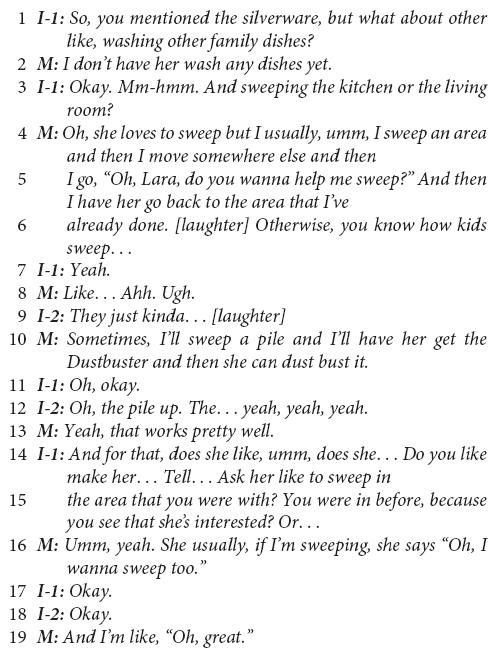


The interviewer’s questions on lines 1 and 3 of the above excerpt are examples of an abbreviated *child-as-agent-helping* prompt, which followed conversationally 26 s after the complete child-as-agent-helping question, “Does [the child] help set or clear the table?” The mother’s responses (line 2; lines 4–6, 10) are archetypical and community-typical examples of a linguistic pivot from *child-as-agent* to *caregiver-as-agent* in reports about the child helping. In the interviewer’s questions, the child is consistently positioned as the agent of the sentence (as in “Does *your child* help…”). In the mother’s utterances, she alters the original structure of the sentence, pivoting from a child-as-agent structure to position herself as the agent in the sentence, as in “*I* don’t have her wash any dishes yet” (line 2).

The mother’s response to the second prompt in this excerpt (lines 4–6) conveys rich information regarding cultural assumptions about children’s motivations to help and everyday family socialization practices regarding the child’s involvement in household work. In line 5, the mother reports that the child “loves to sweep;” however, it is not clear whether the mother is reporting that the child loves to *help* sweep or that the child *loves sweeping*, nor whether their sweeping is connected to involvement in productive household work. However, it is clear by the end of this excerpt that the mother does not love the child’s involvement in sweeping. The mother’s reported frustration (line 8) with the child’s skill or with their manner of becoming involved is reported as a rationale (“otherwise” in line 6) for the mother giving the child “mock work” that occupies the child and distracts them, segregating the child from the productive aspects of the work (see Coppens and Rogoff, in preparation). Managing and limiting the child’s access to the “real work” may be common in this cultural community (Rogoff et al., 1993; Klein and Goodwin, 2013; Klein et al., 2013), and a linguistic pivot away from the child-as-agent suggests the connection of this managerial approach to cultural ethnotheories that de-emphasize the importance of children’s own prosocial initiative.

Of note in this excerpt is the impact of the mother’s linguistic pivots on the interactional trajectory of the interviewer’s questions. The primary interviewer (I-1) is both trained and accustomed to asking about the child’s involvement in household work using the child-as-agent-helping form. However, in lines 14–15, this interviewer becomes conflicted about how to frame subsequent questions with a conversationally appropriate linguistic agent, based on the mothers’ prior pivots. The interviewer begins with a child-as-agent-helping prompt (“umm, does she…”), stops, and begins a new question that deviates from the scripted form of the interview questions (“Do you like make her… Tell… Ask her like to sweep…?”). In an attempt to fit the interview questions to the mothers’ frame of reference, the interviewer takes the mother’s lead in both positioning the mother as the linguistic agent and asking questions about the mother’s management and control of the child’s involvement (i.e., “make,” “tell,” and “ask”). Both adjustments, as well as the interactional context that gave rise to them immediately prior, carry important ethnotheory significance for understanding socialization into helping in the European-American ESE community.

In linguistics, the adoption of an interlocutor’s speech pattern is called *accommodation* (Giles et al., 1991). It is more common for interlocutors to accommodate to the structure of their interlocutors’ speech than it is for them to not accommodate (we have been referring to non-accommodation as a linguistic “pivot” so far). In fact, non-accommodation runs the risk of communicating impoliteness or rudeness (Giles and Gasiorek, 2013). For a speaker to consistently not accommodate to their interlocutor’s speech is the linguistically dispreferred pattern, which usually indicates that what is being communicated is distinct and meaningful. The interviewer’s accommodation, which itself created discomfort as it required the interviewer to deviate from the script, underscores the linguistic preference toward accommodation. The consistent non-accommodation in the European-American ESE mothers’ speech, in which they “pivot,” indicates that the structure to which the mother pivots conveys cultural meaning. When the meaning is not distinct or important for the speaker, the linguistic preference to accommodate the interlocutor’s linguistic structure usually takes precedent.

Another European-American ESE mother’s response below also clearly illustrates linguistic pivots from child-as-agent-helping to caregiver-as-agent.


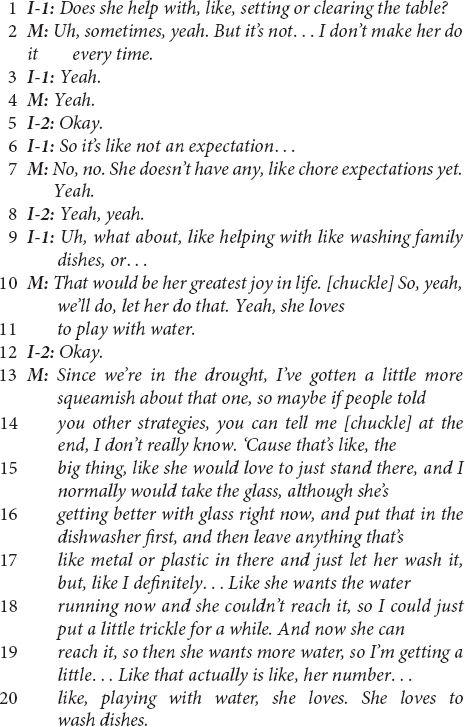


This excerpt illustrates both types of linguistic pivots that were coded in this study. Similar to the previous example, this mother positions her own efforts to *assign* chores to the child and enforce compliance (e.g., line 2, and later clarifications) and her efforts that *permit* the child’s involvement (e.g., “let her do…” on line 10) as agentic rather than continuing with the interviewer’s child-as-agent-helping prompts. Both responses suggest an ethnotheory that the child’s involvement originates with the caregiver’s efforts to organize the child’s access to opportunities to help, rather than with, for example, the child’s initiative (Alcalá and Cervera Montejano, in preparation; Ochs and Kremer-Sadlik, 2013). The responses also suggest an ethnotheory about children’s motivation to help that assumes the necessity of firm caregiver directives (i.e., assignments caregivers “make” the child do).

The above excerpt also illustrates linguistic pivots that reframe the child’s intentions away from helping and toward the child’s assumed efforts to engage in non-help activity – in this excerpt, play (which was predominant in the European-American ESE community for this kind of pivot). In lines 10–11 and lines 19–20, the mother follows descriptions of the child’s engagement in washing dishes with statements that indicate the child’s “greatest joy in life” or “her number [one]” interest is in playing with water. Understood alongside reports that the mother does not expect the child to contribute helpfully, this mother describes the child’s motivation to be involved in household work as a desire to play.

The next excerpt from a European-America ESE mother shows another away-from-helping pivot, its clarity underscored in line 14, a response to a follow-up question.


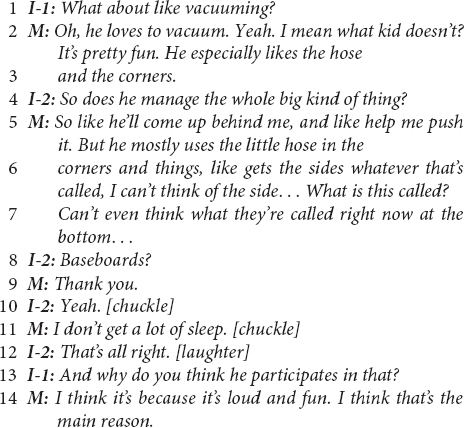


Similar to the previous excerpt, this mother’s response (lines 2–3) immediately pivots away from helping as the purpose of this child’s – indeed, of any child’s – involvement in vacuuming. To be sure, the mother characterizes the child pushing the vacuum with the mother as “help;” however, this is not a motivational attribution. In both linguistic pivots (lines 2–3) and denotational reports (see lines 13–14), the mother makes clear that the child’s central motivation is play.

### “Children-as-Agents-Helping,” a Predominant Linguistic Pattern Among Mothers in a US Mexican-Heritage Community

In this section, we give contextualized examples of the linguistic pattern that overwhelmingly characterized mothers’ responses in the US Mexican-heritage BIW families (as well as for some mothers in the European-American ESE families). We also provide evidence of related features of these mothers’ reports, where children’s assumed motivations to help were discussed in parallel with children’s growing but incomplete skills in contributing.

This first excerpt – in Spanish, then translated to English – from a US Mexican-heritage BIW mother shows the child-as-agent-helping linguistic continuity pattern.


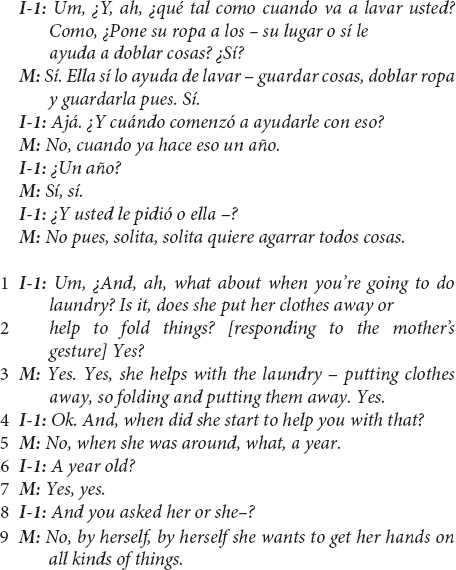


The above excerpt illustrates the linguistic continuity pattern between the interviewer’s question and the mother’s response, continuing to position the child as a helping and helpful agent (question in lines 1–2, response in line 3). Additionally, of note in the above excerpt is a commentary clarifying that the child’s help did not originate developmentally with parents’ efforts and may continue to be driven by the child’s initiative.

This evidence of cultural ethnotheories regarding children’s development of prosocial helping did not solely arise in US Mexican-heritage BIW mothers’ reports of children making contributions; most mothers also maintained continuity with the child-as-agent-helping prompt when reporting that their children did not contribute in a particular task – in the case of one US Mexican-heritage BIW mother:


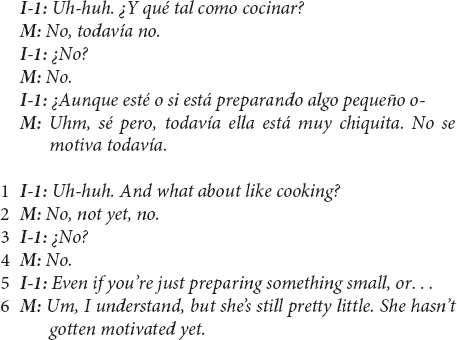


This mother’s responses were straightforward with regard to tasks in which her child did not contribute – she explicitly responded with something similar to “no, not yet” in at least six instances during the interview and seemed to imply such a report in several other responses. Yet, even this brief response suggests important ethnotheoretical information. In line 6, the mother gives a developmental rationale for the child not contributing in stating that the child is “still pretty little” and has not contributed to that “yet,” suggesting that she expects that the child will do so at an older age. The key contextual element in this utterance is the mother’s use of *motivarse*, a reflexive verb in Spanish that translates to “motivating one’s self.” The mother suggests that, in due course, developmentally, children motivate themselves to help with increasingly sophisticated contributions – the family and cultural expectation that children contribute is “in place,” and children are given space to exercise agency in starting to help. The ethnotheoretical assumption presented here is that assigning household work to children, a practice common in middle-class European-heritage communities (Goodnow, 1988; Goodnow and Delaney, 1989; Klein and Goodwin, 2013; Gaskins, 2015; Coppens et al., 2016), is both unnecessary and may conflict with an agency-centered emphasis on children learning to motivate themselves.

The linguistic continuity pattern – overwhelmingly characteristic of mothers’ reports in the US Mexican-heritage BIW community (see Figure 2) – was a common way that mothers in this community reported their children’s involvement such that children’s agency and their assumed intentions or motivations to help were emphasized.

#### Helping While Learning to Contribute: US Mexican-Heritage Mothers’ Negotiations of Skill and Prosocial Intention in Ethnotheories of Toddlers’ Helpfulness

One of the more striking features of the US Mexican-heritage BIW mothers’ reports was that children’s agency-in-helping was asserted in coordination with mothers’ recognition that children were still learning how to contribute. Linguistic patterns provide key evidence for a particular cultural approach to negotiating an apparent socialization paradox in young children’s prosocial development: How can toddlers with relatively low skills be involved in opportunities to contribute, when their helpful intentions outpace their skills?

US Mexican-heritage BIW mothers commonly reported their child’s participation in everyday household work *as help*. The two excerpts below are particularly striking because both mothers appear to distinguish the instrumental contribution of the child’s efforts – *did it contribute?* – from their assumptions regarding what the child was getting involved to do, or the *helpful, prosocial intention* of the effort.


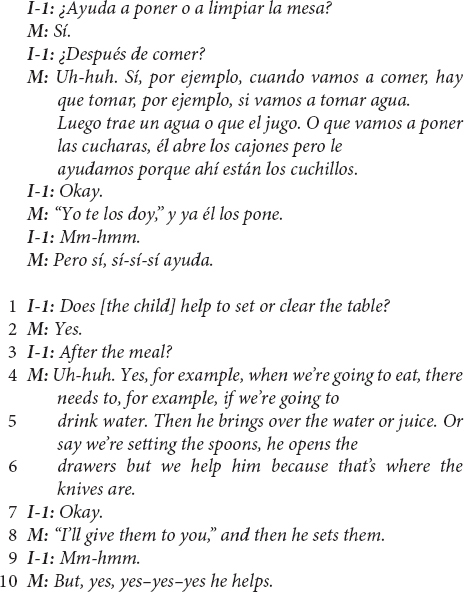


This mother ended reports of the toddler helping in this way (i.e., line 10) – as if to say, “despite what I just described, he really does help!” – four times during just the next 3 min of the interview. The mothers’ efforts to resolve the apparent contrast between the material realities of the child’s contributions (which are small) with the value of those contributions for the mother are clear in the use of repetition to insist that the interviewer be left with the impression that the mother considers the child’s involvement to be *about helping*.

Another US Mexican-heritage BIW mother addresses this contrast more playfully:


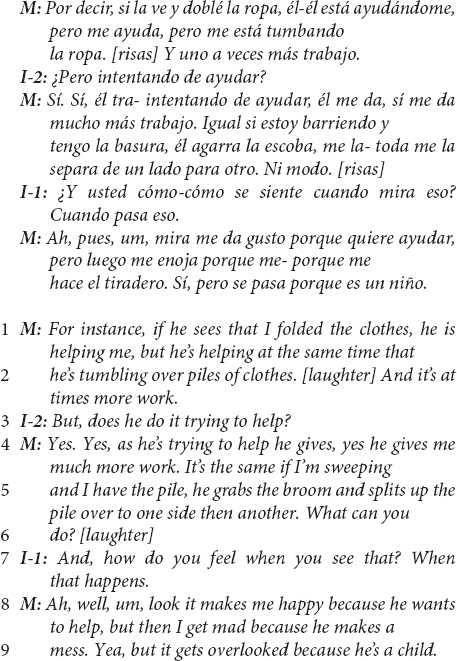


This mother was laughing and smiling throughout this part of the interview, calmly recounting the situation. She followed this excerpt with commentary about how this situation is met with her own guidance and instruction in (but not exclusion from) the household work and that “*está chiquito pero va a aprender”* (“he’s little but he’s going to learn”).

A related linguistic pattern and evidence of cultural ethnotheory is embodied in mothers’ use of the Spanish word, *según*. Nearly half (9/20) of the mothers in the US Mexican-heritage BIW community added this small linguistic feature to reports of their toddler helping at least once during the interview. For example:


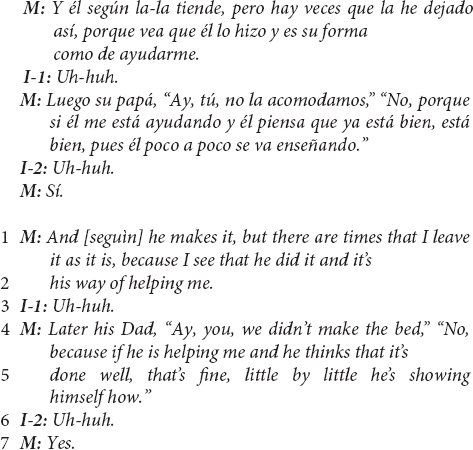


*Según* is a lexically encoded evidential, a linguistic feature that marks the second-hand source of the information being reported (Maldonado and De la Mora, 2015) – in the above excerpt, the information source is the child, or more specifically the mothers’ assumptions about what the child’s perspective is regarding their efforts to make the bed. A common evidential in English is “according to” – e.g., “according to experts…”. *Según* can be approximately translated to “according to,” and it can be used as a pragmatic marker indicating that the information reported may be true for the person being referred to, but that the speaker (i.e., the mother) may or may not align with the information as accurate, revealing a distinct *epistemic stance* (Maldonado and De la Mora, 2015). For example, there is a pragmatically implied difference in a speaker’s epistemic stance (or assessment of the truth claim) in the two sentences: “according to Jim, he helped,” as compared to “Jim helped.”

In this excerpt, it is crucial to accurately understand which information the mother is questioning. This mother explicitly punctuates the child’s prosocial intentions (lines 1–2), which is perhaps necessary because prosociality is not obvious in the outcome of the child’s efforts to make the bed (line 1). The use of *según* evidentially marks the report that the child makes the bed, and the pragmatics of this move are “explained” in the “but there are times that I leave it as is…” that follows – the mother is qualifying the child’s contribution in terms of how well the bed is made.

However, the mother does not attenuate her claim regarding the helpful, prosocial intentions of the child. On the contrary, this mother (a) creates an ethnotheory-significant ideational space by ventriloquizing the child’s father, who she voices as lamenting that the bed has not been made, and then (b) uses the father’s voiced perspective as a foil against which her own perspective can be clarified. Rooted in Bakhtin (1982), this is an understood linguistic pattern whereby individuals, often implicitly, “make their points by positioning themselves with respect to others’ voices, not by speaking directly in their own” (Wortham, 2001b, p. 51). Moreover, these positionings are not merely personal but “provide evidence of how local meanings are shaped by larger institutional contexts” such as ethnotheories (Samuelson, 2009, p. 53; see also Tannen, 2010).

This mother appears to be arguing that, “If I intervene to correct the bed-making (which the child is learning to do better and better), I run the risk of undermining the helpfulness (which the child is already doing well).” *Según*, in this way, is used to distinguish between children skillfully contributing and prosocially helping. Several other US Mexican-heritage BIW mothers used *según* similarly, for example:


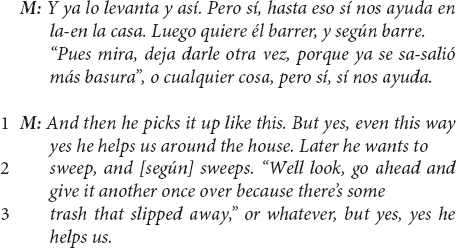


Another US Mexican-heritage BIW mother reported:


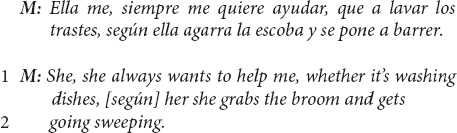


And still another US Mexican-heritage BIW mother:


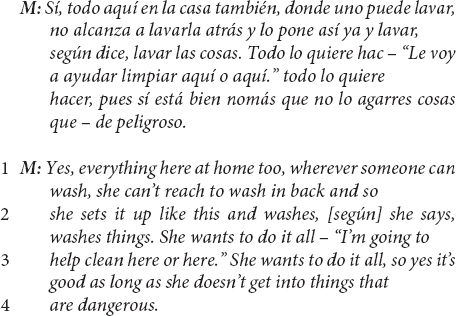


The fact that these mothers use *children’s initiative* in getting involved in everyday household work as evidence of their prosocial intentions to help, even when that involvement may result in a contribution that is imperfect or creates more work for parents, both aligns with mothers’ response continuity with interviewers’ child-as-agent-helping questions and centers children’s agency as a central priority for this community’s cultural approach to socializing children’s development of prosocial helpfulness.

In summary, as shown in Table 1, of the 94 total pivots among European-American ESE mothers, 68 pivots (vs. 4 of 6 pivots in the US Mexican-heritage BIW community) were instances where mothers responded by positioning a caregiver as the linguistic agent instead of the child and 26 pivots (vs. 2 of 6 pivots in the US Mexican-heritage BIW community) replaced helping as the object of the child’s activity with another action (e.g., play). Eighty percent (16/20) of the European-American ESE mothers responded to a child-as-agent-helping question with a pivot that positioned a caregiver as the linguistic agent instead of the child, and 60% (12/20) of European-American ESE mothers replaced helping as the object of the child’s activity with another action. Among US Mexican-heritage BIW mothers, such pivots were far less common (15% or 3/20 and 10% or 2/20, respectively).

## Discussion

This study examined linguistic patterns in mothers’ responses to queries about their children’s help to better understand the *ethnotheories* that may guide how mothers organize and intervene in children’s prosocial engagement. The findings of this study make an important contribution toward understanding cultural differences in parental ethnotheories, family socialization of children’s prosocial development, and ultimately toward understanding possible cultural differences in children’s prosocial helping.

Middle-class European-American mothers in this study frequently did not accommodate the linguistic form of interviewers’ questions about the everyday household activities in which their toddler was involved – questions that positioned the toddler as agentic in helping (Giles et al., 1991). Instead, these mothers often “pivoted” to a linguistic frame that placed their own organizational or motivational efforts as central to the child’s helping (see Figure 1B). Such pivots are meaningful linguistic moves; they function as a bid for revision to the intersubjective ground of meaning between interlocutors. Furthermore, this kind of non-accommodation or “pivot” is unusual in conversational exchange, suggesting that the ethnotheoretical frame to which mothers pivoted is important to their views and values. These finding suggest that middle-class European-American mothers may assume that children’s development of prosocial helping originates with *mothers’* efforts to cultivate helpful dispositions through organizing, managing, and controlling children’s participation in everyday household work (see Lareau, 2000). Such parent-controlled approaches to socializing children’s prosocial helping may be common to middle-class European-American families (Ochs and Kremer-Sadlik, 2013; Coppens et al., 2016), despite the voluntariness of children’s early efforts to get involved. Similarly, the linguistic features of several middle-class European-American mothers’ reports indicated doubt regarding children’s helpful intentions when getting involved with everyday household tasks (see Figure 1C). For example, some mothers responded to questions about their child helping in ways that “pivoted” to reframe the intentions assumed of children in the interviewers’ questions (i.e., that children intended to help) into reports that assumed children’s intentions were to play or do other non-work activities (see also Coppens and Rogoff, in preparation). Some middle-class parents may assume that children are too young to engage with, understand, or be compelled by the needs of others in complex and dynamically coordinated everyday productive endeavors.

In sharp contrast, such “pivots,” whether in regard to children’s agency or children’s assumed intentions to help, were almost entirely absent among US Mexican-heritage mothers (see Figures 2B,C). Linguistic features of these mothers’ reports centered children’s efforts to get involved with ongoing work as driving children’s prosocial development. These findings suggest that US Mexican-heritage mothers “locate” the origins of children’s prosocial development in the early initiatives of young children and may organize their socialization practices in the home to support children’s autonomy, interpersonal responsibility, and collaborative dispositions (Keller et al., 2006; Correa-Chávez et al., 2015; Coppens et al., 2016). Similarly, US Mexican-heritage mothers almost never pivoted away from the linguistic frame that assumed children’s helpful intentions when reporting their toddler’s everyday involvement in family household work (see Figure 2C). On the contrary, these mothers commonly, and at times spontaneously, reported children’s involvement in everyday work *as help* (e.g., “when my child comes into the kitchen to help me”), positioning the child as a contributing member of the family. These findings are consistent with a large body of ethnographic evidence describing indigenous and indigenous-heritage American parents support for children’s autonomy in collaborative efforts and cultural expectations for children’s prosocial helpfulness.

Findings in the US Mexican-heritage BIW community are not entirely consistent with a “relational pathway” of children’s prosocial development found among some communities in India and rural Brazil (Kärtner and Keller, 2012; Köster et al., 2016), with *hierarchical* social relations and adult *assignment* of responsibilities as key elements (see Köster and Kärtner, 2019). Mexican-heritage mothers in this study prioritized children’s initiative in collaborative, more horizontal social relations with children. This emphasis on children’s autonomy exists part and parcel with cultural expectations regarding children’s prosocial helpfulness, which were communicated not as task assignments or requests but through children’s meaningful inclusion in shared endeavors (Rogoff, 2014), creating but not imposing an “inviting horizon” for children’s prosocial development Lee, 1961 (see also Rogoff, 2003; Paradise, 2005). Refined understanding of similarities and differences between these pathways holds strong potential for advancing theories of children’s prosocial development.

The two kinds of linguistic pivots that characterized half of the focal responses by middle-class European-American mothers oppose, respectively, the two core aspects of a widely accepted definition of prosocial helping – “voluntary behavior intended to benefit another” (Eisenberg et al., 2015, p. 114). In so far as such linguistic patterns provide evidence of parental ethnotheories, middle-class European-American parents’ emphasis on their own agency in eliciting children’s help may in practice circumvent children’s opportunities for *voluntary* engagement. Likewise, although less common in these data, middle-class parents’ assumptions that children’s efforts to get involved in everyday household work lack helpful *intentions* may in practice contribute to parents restricting children’s opportunities to learn what helping means and what it entails. To the extent that the linguistic “pivots” found in this study are indicative of ideas that inform parents’ approaches to socializing children’s prosocial behavior, such ethnotheories may inform socialization practices that undermine the very prosocial behaviors they ostensibly aim to develop.

At issue in understanding parental ethnotheories regarding young children’s prosocial helping is not necessarily the accuracy of parents’ appraisals of children’s intentions or motivations. When toddlers get involved in ongoing household work, their motivations are often ambiguous. Is the child trying to help? Are they interested to play, and ambivalent to or unaware of ongoing work? Is the child drawn into everyday work as a way to spend time with a caregiver? Indeed, several motivations may be relevant to children’s interests in getting involved in household work. In contrast with research aiming to discern children’s prior or “underlying” prosocial intentions, our findings raise the possibility that the *assumptions* that parents make about children’s interests, motivations, and intentions play a key role in informing parents’ use of some socialization approaches over others and in organizing the opportunities that children have for collaborative engagement in meaningful family and community work.

The questions of this study have their roots in recent findings suggesting that the often-assumed trajectory of young children’s prosocial development – with toddlers eager to help in everyday household work and older children becoming reluctant or resistant to do so (Rheingold, 1982; Hay and Cook, 2007) – may be a pattern specific to many middle-class communities and generally uncharacteristic of children’s prosocial development in other communities. For example, in a cross-sectional study, Coppens and Rogoff (in preparation) found that middle-class European-American children’s helping at home was limited to just a few low-complexity tasks at both age 2–3 and age 6–7, whereas indigenous-heritage US Mexican children’s help doubled across the same ages. Moreover, middle-class European-American children’s help became less voluntary and more driven by parental directives from age 2–3 to age 6–7, whereas indigenous-heritage US Mexican children’s help became more voluntary and characterized by children’s autonomy and initiative. Other researchers have found similarly pervasive patterns of voluntary helping among children in many non-Western communities (Lancy, 2018).

More research is needed to document and validate evidence of cultural variability in the trajectories of children’s prosocial development, especially focusing on naturalistic settings of children’s everyday lives and on ecologically valid experimental settings (Rogoff et al., 2018). Yet, a parallel question is also pressing: With helpfulness among toddlers seeming to be so pervasive, where do cultural differences in children’s prosocial helping come from?

The importance of understanding parental ethnotheories for developmental questions may stem from their *proleptic* quality (Cole, 1996). Ethnotheories are not simply views that are “held” by parents; they play an important role in how parents interpret children’s actions and how those interpretations inform the guidance that parents provide to children (Goodnow, 1996). For example, Cole (2007) describes British parents’ deployment of culturally stereotypical gender assumptions in talk to and about infants, arguing that “parents’ (purely *ideal*) recall of their [gendered] past and imagination of their child’s future, becomes a fundamental *materialized* constraint on the child’s life experiences in the present” (italics in original, p. 240). Proleptic cultural processes – linking ethnotheories (i.e., *idealized* cultural models) with children’s behaviors and socialization processes (i.e., *material* features of developmental settings) – may be quite common worldwide, even if their content differs from one community to the next. For example, some indigenous-heritage families of Mexico continue the Aztec practice of burying a child’s umbilical cord to connect the child to both gendered community expectations (boys’ and girls’ *ombligos* were buried in different locations, reflecting the tasks in which they were hoped to contribute) as well as to *place* – a physical, cultural, and spiritual location (Rogoff et al., 2014).

Taken as a whole, children’s efforts to engage with ongoing work and the socialization practices that parents use to respond to children’s efforts constitute a “developmental niche” in which prosociality is defined in terms of local expectations for children’s help and is idealized as a developmental goal (Super and Harkness, 1986). For example, if mothers assume that children’s motivation is to play when getting involved with everyday work, they may guide children toward non-work activities that allow the child to play without interrupting household work. Such socialization practices may communicate low expectations that children notice ongoing work, offer to assist, or take responsibility for work around the home. Likewise, ethnotheoretical assumptions that children are inherently motivated to help may lead mothers to provide expanded and supported opportunities for children to learn to be helpful and to learn to collaborate. This kind of trajectory for prosocial development may be supported by expectations that communicate to children, even if implicitly, that their *help* is valued in ongoing productive endeavors, and children may catch on to the meaning of shared work as a result. For example, when asked why they helped at home, Maya children responded, “because I live there” or “*porque el trabajo me lo dice*” (Alcalá et al., in preparation). Although such cultural expectations are rarely examined in studies of children’s helping, they may have a gradual and important impact on children’s prosocial development.

### Extending the Study of Cultural Patterns in Parental Ethnotheories

This study contributes insights into cultural aspects of children’s prosocial development by leveraging linguistic evidence in ways that expand the cultural and methodological basis of psychological and developmental research. In developmental psychology research, interview data are frequently underleveraged for understanding cultural phenomena. Our estimation is that the vast majority of developmental research using interviews attends to the *denotational text* (Wortham et al., 2011) or the “content” reported in the interview – recalled actions or behaviors of the interviewee or others, such as what work parents remember children helping with at home on a regular basis. By extension, asking *cultural* questions of such data requires comparing the denotational reports of informants from different cultural backgrounds. Although interviews can be useful when observational studies are not possible, it is perhaps not surprising that many researchers prefer observational or experimental evidence that does not rely on parents’ recollections.

Interview data often outpace the potential contribution of observations in examining an important aspect of developmental phenomena: parents’ *views*, *ideas*, *beliefs*, and *ethnotheories* regarding, for example, children’s prosocial helping. However, parents’ ideas and ethnotheories are difficult to study even using interviews because (a) the culturally rooted values and assumptions that guide parents’ socialization practices are often experienced implicitly, challenging the viability of methodological approaches that require parents to directly and explicitly report on what they likely experience to be common sense (Geertz, 1975), and (b) the communicative act of asking people questions functions quite differently across cultural communities (Briggs, 1986). Interviewing is never a straightforward process of “collecting” information. Moreover, direct questions to parents about topics with high social desirability across communities – such as the prosocial helpfulness of their children – may yield few cultural differences (de Guzman et al., 2012), complicating attempts to explain otherwise notable cultural differences in children’s prosocial helping (Alcalá et al., 2014; Coppens et al., 2016). Although some researchers have developed innovative approaches for eliciting implicitly held assumptions (e.g., Levy and Hollan, 1998; Keller, 2003; Adair and Kurban, 2019), the empirical study of cultural values and ethnotheories continues to be challenging despite its importance for linking individual and cultural processes.

Our study shows that attention to the *interactional text* of interviews (Wortham et al., 2011) – how language is *put to use* for particular social purposes in both naturally occurring talk and structured research interviews – can reveal cultural expectations and values in ways that are difficult to study otherwise. At times, these interactional features of interviews are difficult to overlook. For example, in a study of mothers’ views on young children’s helping in a community near Guadalajara, México, almost half of the indigenous-heritage mothers that were interviewed resisted an interview question frame about “fairness,” indicating that it was a poor fit for understanding mothers’ views on children’s prosocial responsibilities (Coppens et al., 2016). For example:


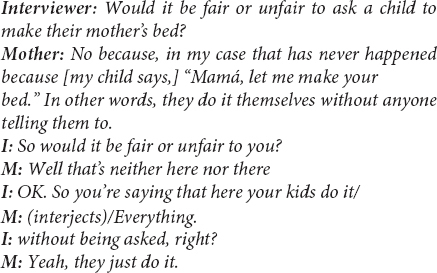


Explicit analytical focus on the social or interactional features of research encounters is rare in psychological research – patterns of interaction between researchers and participants in both interviews and laboratory tasks are often ignored entirely (Packer, 2018). Ignoring such interactional patterns precludes analysis of the social power dynamics present and inevitable in all research, potentially undermining efforts to address issues of equity. Carefully understood, such dynamics can reveal themselves not as “bias” but an important source of evidence regarding the questions and phenomena of interest to psychological researchers (Briggs, 1986), as we have aimed to show in this study. Considerable progress is needed in this area. Our study is one example of systematic and formal approaches to examining and interpreting interactional features of language in research on children’s prosocial development, offering considerable potential for understanding the cultural values, ideas, and ethnotheories that parents draw on in talk about their children and about their children’s prosocial helping. We briefly mention two related efforts by other researchers below.

Keller et al. (2004) used discourse analysis to examine cultural variation in caregivers’ views and ethnotheories regarding what kinds of care are “best” for young children. As with our study, Keller et al. were interested to use the “style” or linguistic form of mothers’ reports to examine cultural perspectives children, families, and parenting – “verbal embodiment, the linguistic means chosen in a given speech-act, can shed light on the implicit aspects of ethnotheories” (p. 294). For example, Keller et al. interpret mothers’ use of “I” statements as evidence of broad alignment with “independent” (vs. “interdependent”) cultural models of parenting. Although we suggest caution in such micro- to macro-interpretative leaps (Wortham, 2012), the analysis nonetheless raises important questions and issues in the study of parental ethnotheories.

Renowned experts in linguistic anthropology, Ochs and colleagues’ research in the *Center on the Everyday Lives of Families* (Klein et al., 2009; Ochs and Kremer-Sadlik, 2013, 2015b) has made valuable contributions to understanding middle-class patterns of children’s everyday prosocial helping. Using detailed linguistic analyses, Ochs and Kremer-Sadlik (2015a) illustrate “coordination troubles” among middle-class families in Los Angeles where parent–child talk both reflects and reifies cultural values, expectations, and ethnotheoretical assumptions. Their data consist of naturally occurring talk in middle-class homes, which also provides evidence of how cultural expectations of responsibility may be communicated to children. For example, after issuing clear directives to two children, imploring their help with cleaning the kitchen table, a middle-class mother “undermines her own authority and rationalization by voicing an imagined ironic disparaging response that she attributes to [one of the children]… Mother: “*deep, creaky voice*” ‘Yeah, right mom.”’ (p. 733). The mothers’ reflexive and ventriloquized response may have the effect of communicating to the child that what is culturally expected is reluctance to help as well as negotiation of the child’s responsibilities to be helpful. This kind of evidence has important implications for the study and understanding of children’s prosocial development in everyday family and community contexts.

These patterns in parental ethnotheories may align with and complement observational findings, providing multilevel insights into cultural features of children’s prosocial helping and development.

### Limitations

Our study was designed to examine theoretically and methodologically significant evidence of parental ethnotheories in two cultural communities. Although we present evidence on the prevalence of several linguistic patterns with ethnotheoretical significance in our sample, much larger samples would be needed to confidently generalize these findings to the entire cultural communities or groups. Our samples are most appropriate for *analytical generalization* or for contributing to theory regarding how culturally variable parental ethnotheories may relate to cultural variability in children’s prosocial helping across development (Firestone, 1993).

To our knowledge, this is the first study in the developmental sciences to connect evidence of linguistic “pivot” patterns to cultural research on parental ethnotheories and the socialization of children’s prosocial development. Further research is needed to understand the interactional features of social science interviews that may permit or preclude this kind of language use. Our study is also the first to connect the Spanish linguistic evidential “*según*” to research on parental ethnotheories of children’s prosocial development. In general, we encourage further study regarding both cultural variability in children’s prosocial helping and examination of cultural processes – linking, for example, parental ethnotheories, socialization practices, and children’s prosocial helping behaviors.

## Conclusion

In conclusion, we offer a cautionary note on the future of comparative cross-cultural research in psychology, including studies of children’s helping and prosocial development. A decade has passed since the publication of Henrich et al. (2010) paper in which the now famous “WEIRD” acronym was coined – Western, educated, industrialized, rich, and democratic. The convenient label has been referred to in over 1,000 published studies (including some of our own prior work). Their analysis contrasted the often-anomalous patterns of performance in experimental research among participants of “WEIRD” cultural backgrounds with the patterns of participants of numerous other cultural backgrounds. The impact of this paper continues to expand, and we strongly agree with its arguments calling for greater sampling diversity across the world’s cultural communities and more cautious and empirically tested generalization on the basis of evidence from this non-majority group. If cultural research on children’s prosocial development and in psychology in general is expanding; these authors share credit with numerous scholars who have, over decades, advocated for such a “turn.”

Nevertheless, the WEIRD label – perhaps due in part to its provocative double entendre – risks reinvigorating the kinds of simplistic, dichotomous cultural comparisons that long-characterized cross-cultural research in psychology. Movement, migration, and cultural hybridity are central to the experiences of a rapidly expanding proportion of the world’s population – if dichotomies such as *individualist versus collectivist* ever adequately characterized cultural contrasts (cf. Rogoff, 2003), their validity should be under increased scrutiny. Our caution at present is that explicit or implied dichotomous contrasts between “WEIRD” and “non-WEIRD” cultural groups do not productively advance the field of cultural research in children’s development. As we have endeavored in this paper, when researchers take care to explore family practices and patterns with attention to cultural context, cultural contrasts and, comparisons can be explored in ways that are neither reductive nor dichotomous. Thomas (1958) cautioned similarly with regard to research on Cherokee cultural values:

What really bothers me methodologically is that Cherokees sound so much like other American Indians. You could, almost, substitute the word Cherokee for much of the material present on Navajo values or Chippewa, and so on, around the country. We haven’t the terms to really describe this behavior and thus differentiate, except at a gross level.… [Are] we really seeing American Indians at even this gross level? Are we seing [*sic*] tribal societies? Or are we just seeing the European in negative? (p. 25)

This study responds to the need for both within- and across-community sophistication in cultural research in psychology by drawing on the ethnographically informed methodological and analytic traditions of linguistic anthropology. In doing so, we highlight a collaborative, prosocial socialization paradigm common among many indigenous-heritage Mexican families in and outside of the United States as an inspiring model of cultural strengths for organizing opportunities for children, alongside adults, to learn and develop prosocially by contributing, collaborating, and belonging (Coppens et al., 2014; Rogoff et al., 2017). This model provides a promising horizon for revising and expanding theories of children’s prosocial development.

## Data Availability Statement

The data used in this study are held in encrypted online storage by the first author, and inquiries regarding the data should be addressed to the first author. Due to special circumstances of confidentiality required for some of the families in this study, data cannot be made publicly available.

## Ethics Statement

The studies involving human participants were reviewed and approved by the University of California, Santa Cruz Institutional Review Board. The participants provided their written informed consent to participate in this study.

## Author Contributions

ADC contributed study design and data collection. ADC and AIC contributed early conceptualization and analysis. ADC and LA coded the data. ADC wrote the first draft of the manuscript. ADC, AIC, and LA wrote sections of the manuscript. All authors contributed to manuscript revision, read, and approved the submitted version.

## Conflict of Interest

The authors declare that the research was conducted in the absence of any commercial or financial relationships that could be construed as a potential conflict of interest.
